# Cardiovascular health and risk of hospitalization with COVID-19: A Mendelian Randomization study

**DOI:** 10.1177/20480040211059374

**Published:** 2021-11-19

**Authors:** Marina Cecelja, Cathryn M. Lewis, Ajay M. Shah, Phil Chowienczyk

**Affiliations:** 1 Department of Clinical Pharmacology, King's College London British Heart Foundation Centre, School of Cardiovascular Medicine & Sciences, St Thomas’ Hospital, London, UK; 2 Social, Genetic and Developmental Psychiatry Centre, Institute of Psychiatry, Psychology and Neuroscience, 4616 King's College London, London, UK; 3 Department of Medical and Molecular Genetics, 405987 Faculty of Life Sciences and Medicine, King's College London, UK; 4 School of Cardiovascular Medicine & Sciences, Department of Cardiology, King's College London British Heart Foundation Centre, London, UK

**Keywords:** COVID-19, cardiovascular risk factors, mendelian randomization

## Abstract

**Background:**

Susceptibility to and severity of COVID-19 is associated with risk factors for and presence of cardiovascular disease.

**Methods:**

We performed a 2-sample Mendelian randomization to determine whether blood pressure (BP), body mass index (BMI), presence of type 2 diabetes (T2DM) and coronary artery disease (CAD) are causally related to presentation with severe COVID-19. Variant-exposure instrumental variable associations were determined from most recently published genome-wide association and meta-analysis studies (GWAS) with publicly available summary-level GWAS data. Variant-outcome associations were obtained from a recent GWAS meta-analysis of laboratory confirmed diagnosis of COVID-19 with severity determined according to need for hospitalization/death. We also examined reverse causality using exposure as diagnosis of severe COVID-19 causing cardiovascular disease.

**Results:**

We found no evidence for a causal association of cardiovascular risk factors/disease with severe COVID-19 (compared to population controls), nor evidence of reverse causality. Causal odds ratios (OR, by inverse variance weighted regression) for BP (OR for COVID-19 diagnosis 1.00 [95% confidence interval (CI): 0.99–1.01, P = 0.604] per genetically predicted increase in BP) and T2DM (OR for COVID-19 diagnosis to that of genetically predicted T2DM 1.02 [95% CI: 0.9–1.05, P = 0.927], in particular, were close to unity with relatively narrow confidence intervals.

**Conclusion:**

The association between cardiovascular risk factors/disease with that of hospitalization with COVID-19 reported in observational studies could be due to residual confounding by socioeconomic factors and /or those that influence the indication for hospital admission.

## Introduction

Clinical manifestations of COVID-19 vary from asymptomatic infection, mild upper respiratory tract illness to viral pneumonia which may progress to respiratory failure and death in severe cases.^1^ Risk factors that predispose to increased risk of hospitalization with COVID-19 include a predominance of risk factors for and presence of cardiovascular disease: hypertension, type 2 diabetes mellitus (T2DM), obesity and presence of coronary artery disease (CAD).^2–4^Furthermore, once hospitalized, presence of cardiovascular risk factors and disease has been shown to increase the risk of death.^5–7^ However, whether there is a causal association between these factors and severity of COVID-19 is unknown since observational studies do not determine direction of causality and are limited by residual and unknown confounding. Mendelian randomization (MR) is a useful tool which uses genetic variants to investigate causal associations between risk factors and disease outcome in observational data.^8^ Since genetic variants are inherited randomly, MR is akin to performing a randomized controlled trial. The aim of the present study was to investigate the association between cardiovascular risk factors and presence of cardiovascular disease and the susceptibility to severe COVID-19 by performing a two-sample MR analysis using the most recently published genome-wide association and meta-analysis studies with publicly available summary-level genome wide association studies (GWAS). In addition, we investigated reverse causality between genes associated with COVID-19 and cardiovascular risk factors and CAD to investigate whether presence of severe COVID-19 may lead to development of cardiovascular disease.^9^

## Methods

Two-sample MR was used to investigate the hypothesized causal effect of cardiovascular risk factors and CAD on susceptibility to and severity of COVID-19. Summary-level data were collected from genome-wide association studies (GWAS) where the variant-exposure associations were estimated in one dataset and variant-outcome associations were estimated from another.

### Variant-Exposure Associations

Exposures considered in this analysis included blood pressure (BP), T2DM, obesity (measured as body mass index, BMI) and CAD. Variant-exposure associations were determined from most recently published genome-wide association and meta-analysis studies with publicly available summary-level GWAS data extracted manually.^10–13^ The following data was extracted for each genetic instrumental variable (IV): the effect allele, beta value (or odds ratio (OR)), standard error (or 95% confidence interval (CI) of the OR), SNP ID (or SNP position) and p-value. Variant harmonization was performed manually to check that genetic variants were appropriately orientated across the datasets. Where there was discrepancy between summary statistics for exposure and outcome variables, the sign of beta values for COVID-19-SNP associations were reversed. We extracted all available summary results for 984 BP variants reported in a GWAS meta-analysis of 454,577 (45.8% male) individuals (Evangelou et al.,^10^ supplementary tables 4, 18 and 24). Summary results for 403 type 2 diabetes related variants were extracted from a GWAS of 898,130 individuals (Mahajan et al.,^11^ supplementary Table 2). For BMI, data for 546 SNPs were extracted from a meta-analysis including 806,834 participants (Pulit et al.^12^^)^ and for CAD summary data for 65 variants were extracted from a GWAS of 185,000 CAD cases and controls (Nikpay et al.,^13^ supplementary tables 2 and 4). MR analysis was performed using only SNPs associated with the exposure variable with a p-value < 1  ×  10^−8^ for CAD. For categorical exposures (e.g. presence or absence of T2DM), beta coefficients were taken as the log of the reported odds ratio (OR) and 95% confidence intervals (CI) of the OR converted into a standard using the formula: standard error = (ln(upper 95% CI) - ln(lower 95% CI))/1.95996*2.

### Variant-Outcome Associations

GWAS meta-analysis of patients with a laboratory confirmed diagnosis of COVID-19 and with severity requiring hospitalization were obtained from recently released data from www.covid19hg.org/ (released 18 January 2021 round 5). This analysis is based on a mostly European (55%) and US (28%) population. However, participants from Asia, Australia, Middle-East and Africa were also included (ref: https://doi.org/10.1038/s41431-020-0636-6). Only variants with imputation quality of over 0.6 were retained. Analysis was performed with cases (n = 5582) classified as those diagnosed with COVID-19 and need for hospitalization versus the remainder of the population not defined as a case (controls, n = 709,010). Studies included in this analysis were BioMe, FinnGen, Genes & Health, Lifelines Global Screening Array, Lifelines CytoSNP, Netherlands Twin Registry, Partners Healthcare Biobank and UK Biobank. Data from all studies was included.

### Statistical Analysis

To investigate the causal association between cardiovascular risk factors to hospitalization with COVID-19 we performed two-sample MR with STATA software and the command *mrrobust* using multiple instruments. The association between IV and outcome was assessed using inverse-variance weighted (IVW) regression models as the primary analysis. This is equivalent to a weighted regression of the SNP-outcome estimate on SNP-exposure estimate with the intercept constrained to zero. This method assumes no horizontal pleiotropy. The causal estimate of the IVW analysis is the increase in severity of COVID-19 per unit change in the genetically predicted exposure. Following MR analysis, beta coefficients were converted back into OR using the *lincom* command in STATA. We performed a sensitivity analysis using a weighted median method which is less sensitive to outliers and, in order to account for any potential violation of the assumption of no horizontal pleiotropy, we also used MR-Egger regression. We tested for the presence of directional pleiotropy by testing significance of the intercept in the MR-Egger analysis.^8^

#### Reverse Causality

We additionally assessed reverse causality to determine whether severity of COVID-19 associated with cardiovascular risk factors using two-sample MR performed using summary-level GWAS data available from the platform MR-Base (http://www.mrbase.org).^14^ As above, severity was defined as laboratory confirmed COVID-19 infection and hospitalized for COVID-19. Studies included in this analysis were BioMe, FinnGen, Genes & Health, Lifelines Global Screening Array, Lifelines CytoSNP, Netherlands Twin Registry, Partners Healthcare Biobank and UK Biobank. Data from all studies was included. For this analysis the IV were built considering GWAS suggestive SNPs for COVID-19 severity (P < 5 × 10^−8^, n = 493). Clumping was used to prune SNPs for linkage disequilibrium and thus not all SNPs were included in the final analysis. If a SNP was absent in the summary GWAS statistics, a proxy SNP in high LD with r^2^ ≥ 0.80 was used where available. However, if this was not successful, the SNP was excluded. The association between IV and outcome was assessed using IVW regression models, weighted median method and MR-Egger. Leave-one-out sensitivity was performed to exclude the possibility of one SNP having a large effect on the overall results. To determine whether risk of COVID-19 associated SNPs are associated with BP (n = 317,754), BMI (n = 336,107) and T2DM (n = 336,473) outcome data from UK Biobank were selected from MR-Base. To determine whether risk of COVID-19 associated SNPs are associated with CAD (n = 184,305) outcome data from CARDoGRAMplusC4D cohort were selected from MR-Base.

## Results

### Risk factors for severity to covid-19

**Figure 1.**
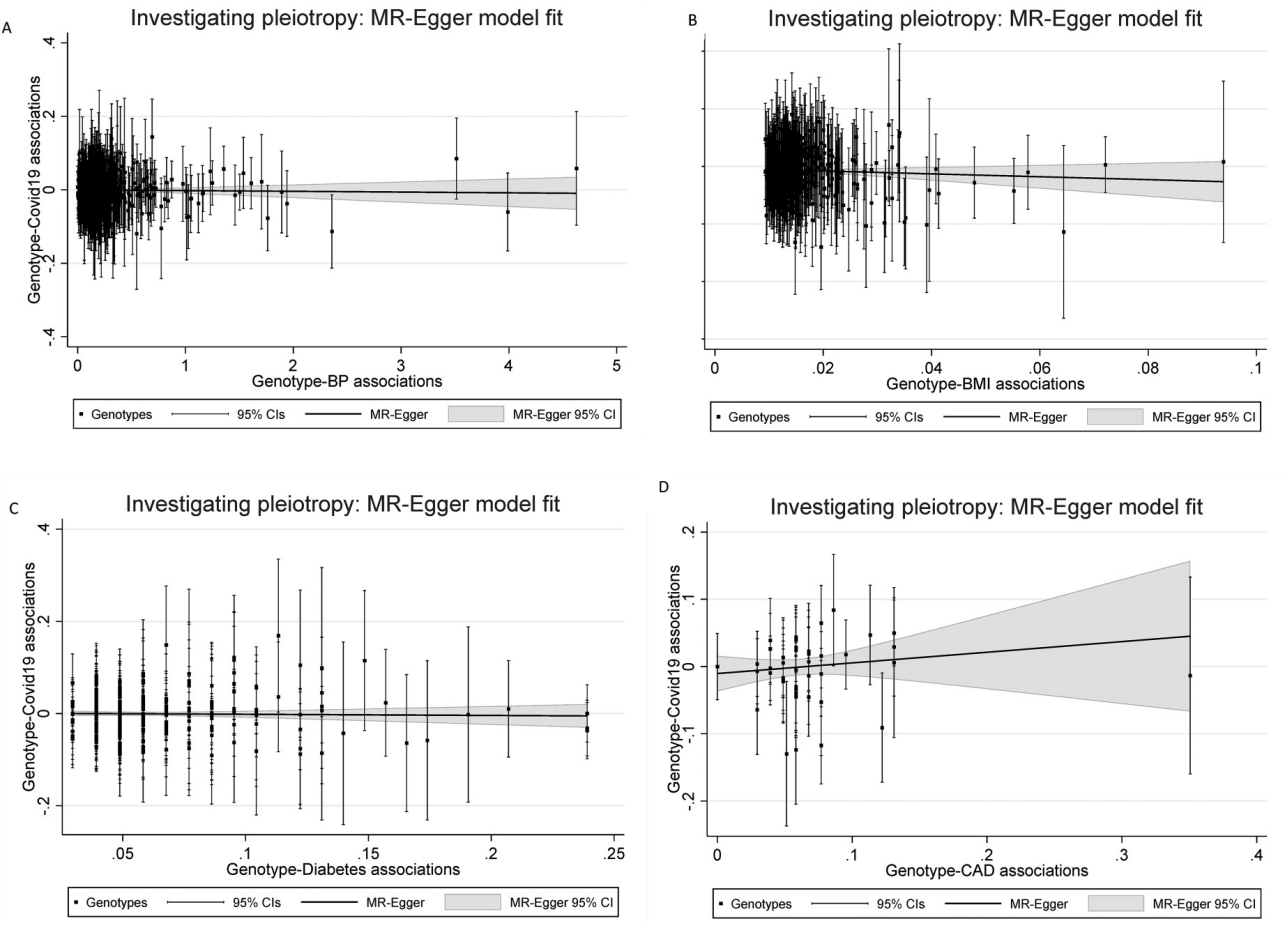
Scatter plot of genetic associations with the outcome (y axis) against genetic associations with the exposure (x axis). A) Effect of genetically predicted BP on risk of susceptibility with COVID-19. B) Effect of genetically predicted BMI on risk of susceptibility with COVID-19. C) Effect of T2DM on risk of susceptibility with COVID-19. D) Effect of CAD on risk of susceptibility with COVID-19. The slope of the regression line represents the causal association.

When examining the association of cardiovascular risk factors/disease to risk of hospitalization with COVID-19, 639, 413, 270 and 45 SNPs were available for BP, BMI, T2DM and CAD, respectively, in summary-level COVID-19 severity outcome data. The main MR results are shown in Table 1. For BP we found no evidence of a causal association with COVID-19 hospitalization: OR of 1.00 (95% CI: 0.99-1.01, P = 0.604, Figure 1A) per unit mmHg increase in genetically predicted BP. IVW regression suggested a negative causal effect of BMI on COVID-19 hospitalization with an estimated OR of 0.66 (95% CI: 0.55-0.80, P < 0.001, Figure 1B) for COVID-19 per unit increase in genetically predicted BMI. However, this analysis was not supported by Egger regression estimates (MR-Egger beta coefficient = -0.25, P = 0.268). For the other factors we found no evidence of a causal association with COVID-19 hospitalization: OR for COVID-19 hospitalization to that of genetically predicted T2DM 0.99 (95% CI: 0.93-1.05, P < 0.927, Figure 1C) and OR for COVID-19 hospitalization to that of genetically predicted CAD 1.02 OR (95% CI: 0.85–1.23, P < 0.795, Figure 1D). The weighted median regression and MR-Egger regression estimates were consistent with these findings.

**Table 1.** Mendelian randomization with cardiovascular risk factors as the exposure and Covid-19 hospitalization as the outcome.

Exposure	Method	beta (SE)	OR (95% CI)	P-value
Hospitalized for COVID-19				
BP	IVW	−0.01 (0.004)	1.00 (0.99–1.01)	0.604
	MR Egger	0.01 (0.005)		0.692
BMI	IVW	−0.41 (0.10)	0.66 (0.55–0.80)	<0.001
	MR Egger	−0.25 (0.23)		0.268
T2DM*	IVW	−0.01 (0.03)	0.99 (0.93–1.05)	0.927
	MR Egger	−0.03 (0.07)		0.717
CAD*	IVW	0.02 (0.09)	1.02 (0.85–1.23)	0.795
	MR Egger	0.16 (0.19)		0.406

SE  =  standard error; OR  =  odds ratio; CI  =  confidence interval; BP  =  blood pressure; BMI  =  body mass index; T2DM  =  type 2 diabetes mellitus; CAD  =  coronary artery disease; IVW  =  inverse variance weighted. *Note that T2DM and CAD were binary variables and effect sizes are thus log-odds ratios.

### Reverse causality

Of the 493 alleles associated with Covid-19 hospitalization, 4 alleles were included in the 2-sample MR analysis with BP as the exposure. The main MR results are shown in Table 2 and Supplement Figure 1. We found no evidence for an association between COVID-19 hospitalizations and BP, T2DM or CAD. Results were similar when using weighted median regression, MR-Egger and IVW regression. We found some evidence of a negative causal association between COVID-19 hospitalization and BMI with an estimated beta coefficient of −0.02 (standard error of 0.01, P < 0.05, Supplement Figure 1C). However, these findings were not supported by weighted median regression (P = 0.087) and MR-Egger (P = 0.500) analysis. The MR-Egger regression estimate was not significantly greater than zero suggesting no evidence of horizontal pleiotropy.

**Table 2.** Mendelian randomization (MR) with hospitalization for COVID-19 as the exposure and cardiovascular risk factors as the outcome.

Outcome	Method	beta	SE	P-value
Systolic BP	IVW	−0.001	0.02	0.6681
	Weighted median	0.000	0.01	0.9298
Diastolic BP	IVW	−0.001	0.008	0.1297
	Weighted median	−0.01	0.008	0.1805
Body mass index	IVW	−0.02	0.009	<0.05
	Weighted median	−0.01	0.009	0.1008
Diabetes mellitus	IVW	−0.002	0.002	0.1825
	Weighted median	−0.002	0.002	0.1116
Coronary artery disease	IVW	−0.006	0.05	0.2448
	Weighted median	−0.03	0.03	0.3434

SE  =  standard error; BP  =  blood pressure; IVW  =  inverse variance weighted.

## Discussion

**Figure 2.**
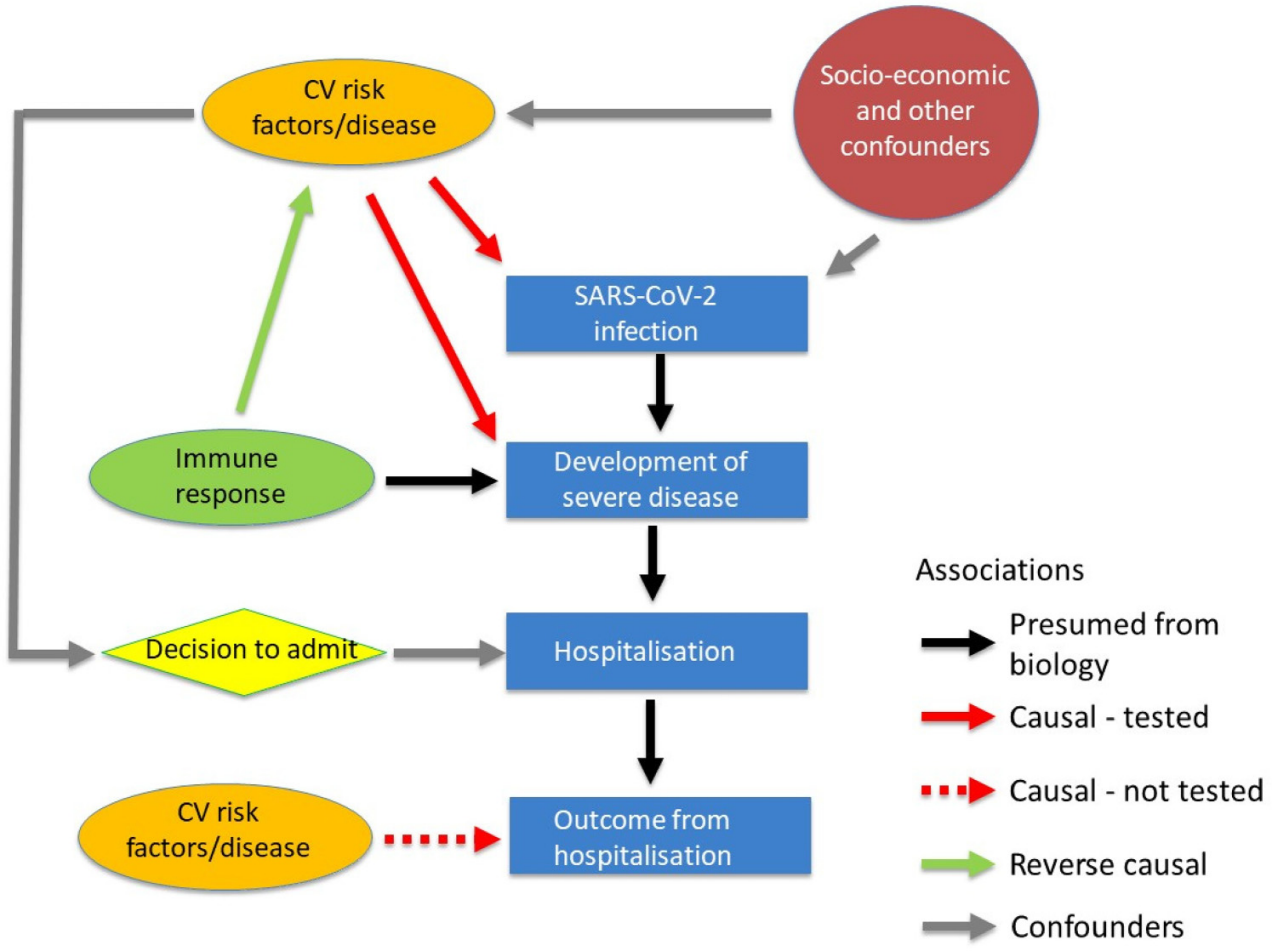
Cardiovascular risk factors/disease (CVRD) and hospitalisation with COVID-19 may be causally or reverse causally associated or the association may arise through confounding. It is assumed that hospitalisation results from infection from SARS-CoV-2 and developing severe COVID-19 (black arrows). CVRD may be causally related to hospitalisation through increased susceptibility to infection and/or contributing to increased severity of COVID-19 (solid red arrows). This causal relationship is tested by the current Mendelian randomization. On the assumption that severity of COVID-19 depends in part on the immune response a reverse causal association between hospitalisation with COVID-19 and CVRD could occur (Green arrow). This is also tested by the current Mendelian randomisation. Socioeconomic (and other) factors that predispose to infection by SARS-CoV-2 and also to CVRD could cause an association through confounding (grey arrows). The decision to admit patients with COVID-19 may be influenced by presence of CVRD and cause an association through confounding. Not tested in the present Mendelian randomization is whether CVRD may causally influence survival after hospitalisation with COVID-19.

Most cardiovascular disease is thought to arise on a background of endothelial cell dysfunction mediated directly or indirectly by risk factors for cardiovascular disease.^15^ SARS-CoV-2 enters endothelial cells as well as epithelial cells and many of the complications of COVID-19 are thought to be due to an endotheliitis.^1^ A causal link between cardiovascular risk factors/disease and COVID-19 is therefore entirely plausible with CVD risk or disease increasing the susceptibility to invasion of endothelial cells by SARS-CoV-2 and/or exacerbating the resultant endothelial cell injury. In the present study, using MR, we found no consistent evidence of a causal (or reverse causal) association of the risk of hospitalization with COVID-19 with major CVD risk factors (BP, BMI and T2DM) or with the presence of CAD as predicted by genetic variants. Whilst the confidence limits for the causal effect of BMI and CAD on COVID-19 severity were wide and we cannot exclude clinically significant causal effects, those for BP and T2DM were relatively narrow. This raises the question of whether the associations of COVID-19 outcomes with these risk factors that have been observed in observational studies may have arisen through an unmeasured confounder. Risk of hospitalization with COVID-19 is a product of risk of infection with SARS-Cov2 and that of infection resulting in disease sufficiently severe to require hospital admission. Risk of infection may be associated with socio-economic factors that cluster with cardiovascular disease whilst hospitalization will be to some extent dependent on an individual patient's perception of severity and physician decision to admit may be dependent on risk factors and co-morbidities (Figure 2). Thus, socioeconomic and admission decisions may be important confounders. Genetically (or otherwise) determined immune response could be the main causal factor determining severity of COVID-19. A non-causal association of COVID-19 outcomes with CVD risk factors could then occur due to this feature of immune response amplifying effects of environmental/genetic factors that increase the prevalence of risk factors, particularly BP and T2DM, but these are not in themselves causal factors for COVID-19 outcomes. In this respect it is notable that altered immune function has been implicated in both hypertension^16^ and T2DM.^17^ However, in this case we would expect a reverse causal effect of risk factors for COVID-19 hospitalization influencing genetically predicted cardiovascular disease which we did not observe.

With the exception of the lack of association with genetically predicted BMI, our findings are consistent with a recent MR study investigating the association between lipids, systolic blood pressure and type 2 diabetes with severity of COVID-19 that used summary genetic association estimates for risk of severe COVID-19 with respiratory failure from a separate European cohort to ours.^18^ This study did find evidence of a causal association between fat distribution and COVID-19 outcomes. This difference could be because the SNPs used as instrumental variables were those identified to associate with waist-to-hip ratio adjusted for BMI (rather than BMI itself as in our study). Our findings, apart from those relating to BMI, are also consistent with a MR study investigating the association of various cardiometabolic traits including diabetes, fasting glucose, low-density lipoprotein cholesterol (LDL-cholesterol), triglycerides, BP and presence of CAD and stroke.^19^ The authors did find a causal association between BMI and COVID-19 outcomes but not with BMI adjusted waist-hip ratio. However, the authors used summary statistics from only 75 SNPs whereas we used >400 SNPS identified to associate with BMI and data from the most recent meta-analysis GWAS for COVID-19.^19^ Similarly, other studies have found inconsistent findings between BMI and LDL-cholesterol with COVID-19 outcomes.^20–22^

It is important to note that our study did not address a causal association of genetically predicted cardiovascular risk factor/disease with survival from COVID-19 once hospitalized (because of the lack of GWAS data on this outcome). Several large studies have shown that, once hospitalized, presence of cardiovascular risk factors and disease is associated with increased mortality.^5–7^ Further studies will be required to determine whether this association is causal.

### Strengths and Limitations

The present study has a number of strengths. The IV chosen for BP, T2DM and CAD were from the most recent summary level statistics with p-values < 10^−8^, protecting against weak instrument bias. However, we do recognize that a larger number of SNPs have been identified to associate with different indices of obesity and fat distribution, and that the instrumental variables chosen for the causal association between BMI and COVID-19 may be subject to weak instrument bias which may bias our results towards the null. By using two-sample MR we were also able to investigate reverse causality of COVID-19 outcomes on CVD risk factors and CAD. There may have been differences in populations demographics from which the summary level statistics in the exposure and outcome samples were obtained. However, the effect of this is likely to be limited as variant-exposure and variant-outcome association were derived from mostly European populations. Since COVID-19 clusters are mainly in cities we cannot exclude the possibility that the control group is not a representative source population and overlap of populations from which the summary level statistics are obtained may bias the association estimates. However, overlap of populations was only observed for the reverse causality analysis and the bias introduced would be more likely to produce a false-positive rather than the null result we observed. There are limitations in the interpretation of the genetically predicted outcome measures we used since most asymptomatic subjects with COVID-19 will not receive a diagnosis and hospitalization as measure of severity limited because patients with co-morbidities may be more likely to be hospitalized because risk of adverse outcomes is thought to be higher.

## Conclusion

The present MR study found no consistent evidence of a causal or reverse-causal association between major genetically predicted CVD risk factors (BP, BMI and T2DM) and presence of CAD with risk of hospitalization with COVID-19. The association between CVD risk factors and risk of hospitalization with COVID-19 observed in observational studies could be due to residual confounding by socioeconomic factors or factors related to admission decisions. Our study does not address a causal relation between cardiovascular risk factors and severity of disease once hospitalized with COVID-19.

## Supplemental Material

sj-docx-1-cvd-10.1177_20480040211059374 - Supplemental material for Cardiovascular health and risk of hospitalization with COVID-19: A Mendelian Randomization study Click here for additional data file. Supplemental material, sj-docx-1-cvd-10.1177_20480040211059374 for Cardiovascular health and risk of hospitalization with COVID-19: A Mendelian Randomization study by Marina Cecelja, Cathryn M. Lewis and Ajay M. Shah, Phil Chowienczyk in JRSM Cardiovascular Disease

## References

[1] Stawicki SP Jeanmonod R Miller AC , et al. The 2019-2020 novel coronavirus (severe acute respiratory syndrome coronavirus 2) pandemic: a joint American college of academic international medicine-world academic council of emergency medicine multidisciplinary COVID-19 working group consensus paper. J Glob Infect Dis 2020; 12: 47–93. 32773996 10.4103/jgid.jgid_86_20 PMC7384689

[2] Zhou F Yu T Du R , et al. Clinical course and risk factors for mortality of adult inpatients with COVID-19 in wuhan, China: a retrospective cohort study. Lancet 2020; 395: 1054–1062. 32171076 10.1016/S0140-6736(20)30566-3 PMC7270627

[3] Driggin E Madhavan MV Bikdeli B , et al. Cardiovascular considerations for patients, health care workers, and health systems during the COVID-19 pandemic. J Am Coll Cardiol 2020; 75: 2352–2371. 32201335 10.1016/j.jacc.2020.03.031 PMC7198856

[4] Shi Y Yu X Zhao H , et al. Host susceptibility to severe COVID-19 and establishment of a host risk score: findings of 487 cases outside wuhan. Crit Care 2020; 24: 108. 32188484 10.1186/s13054-020-2833-7 PMC7081524

[5] Loffi M Piccolo R Regazzoni V , et al. Coronary artery disease in patients hospitalised with coronavirus disease 2019 (COVID-19) infection. Open Heart 2020; 7: 7. 10.1136/openhrt-2020-001428 PMC7684763 33229434

[6] Collard D Nurmohamed NS Kaiser Y , et al. Cardiovascular risk factors and COVID-19 outcomes in hospitalised patients: a prospective cohort study. BMJ Open 2021; 11: e045482. 10.1136/bmjopen-2020-045482 PMC7902321 33619201

[7] Silverio A Di Maio M Citro R , et al. Cardiovascular risk factors and mortality in hospitalized patients with COVID-19: systematic review and meta-analysis of 45 studies and 18,300 patients. BMC Cardiovasc Disord 2021; 21: 23. 33413093 10.1186/s12872-020-01816-3 PMC7789083

[8] Burgess S Davey Smith G Davies NM , et al. Guidelines for performing mendelian randomization investigations. Wellcome Open Res 2019; 4: 186. 32760811 10.12688/wellcomeopenres.15555.1 PMC7384151

[9] The European Society for Cardiology. ESC Guidance for the Diagnosis and Management of CV Disease during the COVID-19 Pandemic. https://www.escardio.org/Education/COVID-19-and-Cardiology/ESCCOVID-19-Guidance. (Last update: 10 June 2020).

[10] Evangelou E Warren HR Mosen-Ansorena D , et al. Genetic analysis of over 1 million people identifies 535 new loci associated with blood pressure traits. Nat Genet 2018; 50: 1412–1425. 30224653 10.1038/s41588-018-0205-x PMC6284793

[11] Mahajan A Taliun D Thurner M , et al. Fine-mapping type 2 diabetes loci to single-variant resolution using high-density imputation and islet-specific epigenome maps. Nat Genet 2018; 50: 1505–1513. 30297969 10.1038/s41588-018-0241-6 PMC6287706

[12] Pulit SL Stoneman C Morris AP , et al. Meta-analysis of genome-wide association studies for body fat distribution in 694 649 individuals of european ancestry. Hum Mol Genet 2019; 28: 166–174. 30239722 10.1093/hmg/ddy327 PMC6298238

[13] Nikpay M Goel A Won HH , et al. A comprehensive 1,000 genomes-based genome-wide association meta-analysis of coronary artery disease. Nat Genet 2015; 47: 1121–1130. 26343387 10.1038/ng.3396 PMC4589895

[14] Hemani G ZJ Wade KH Laurin C , et al. The MR_base platform supports systematic causal inference across the human phenome. eLife 2018. 10.7554/eLife.34408 PMC5976434 29846171

[15] Versari D Daghini E Virdis A , et al. Endothelial dysfunction as a target for prevention of cardiovascular disease. Diabetes Care 2009; 32: S314–S321. 19875572 10.2337/dc09-S330 PMC2811443

[16] Vasdev S Stuckless J Richardson V . Role of the immune system in hypertension: modulation by dietary antioxidants. Int J Angiol 2011; 20: 189–212. 23204821 10.1055/s-0031-1288941 PMC3331645

[17] Wu C Chen X Shu J , et al. Whole-genome expression analyses of type 2 diabetes in human skin reveal altered immune function and burden of infection. Oncotarget 2017; 8: 34601–9. 28427244 10.18632/oncotarget.16118 PMC5470994

[18] Mark P Gkatzionis A Walker V , et al. Cardiometabolic traits, sepsis and severe covid-19 with respiratory failure: a Mendelian randomization investigation 2020. 10.1161/CIRCULATIONAHA.120.050753 PMC7594537 32966752

[19] Leong A Cole JB Brenner LN , et al. Cardiometabolic risk factors for COVID-19 susceptibility and severity: a mendelian randomization analysis. PLoS Med 2021; 18: e1003553. 33661905 10.1371/journal.pmed.1003553 PMC7971850

[20] Ponsford MJ Gkatzionis A Walker VM , et al. Cardiometabolic traits, sepsis, and severe COVID-19: a mendelian randomization investigation. Circulation 2020; 142: 1791–1793. 32966752 10.1161/CIRCULATIONAHA.120.050753 PMC7594537

[21] Aung N Khanji MY Munroe PB , et al. Causal inference for genetic obesity, cardiometabolic profile and COVID-19 susceptibility: a mendelian randomization study. Front Genet 2020; 11: 586308. 33262790 10.3389/fgene.2020.586308 PMC7686798

[22] Lorincz-Comi N Zhu X . Cardiometabolic risks of SARS-CoV-2 hospitalization using mendelian randomization. Sci Rep 2021; 11: 7848. 33846372 10.1038/s41598-021-86757-3 PMC8042041

