# Supplementary material for: Cardiovascular health and risk of hospitalization with COVID-19: A Mendelian Randomization study
Source: JRSM Cardiovasc Dis. 2021 Nov 19;10:20480040211059374. doi: 10.1177/20480040211059374 (PMC8619738; doi:10.1177/20480040211059374)
Supplement: sj-docx-1-cvd-10.1177_20480040211059374 - Supplemental material for Cardiovascular health and risk of hospitalization with COVID-19: A Mendelian Randomization study [file sj-docx-1-cvd-10.1177_20480040211059374.docx]

**SUPPLEMENT**

**Supplement Figure 1** Forest plot of 2-sample Mendelian randomization (MR) (A) with systolic blood pressure (SBP); (B) with diastolic blood pressure (DBP); C) with body mass index (BMI); D) with diabetes mellitus (DM) and E) with coronary artery disease (CAD) as the outcome.

A)


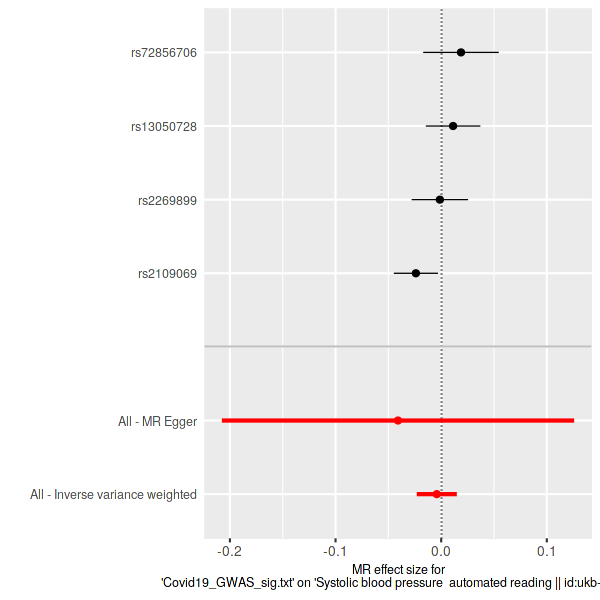


B)


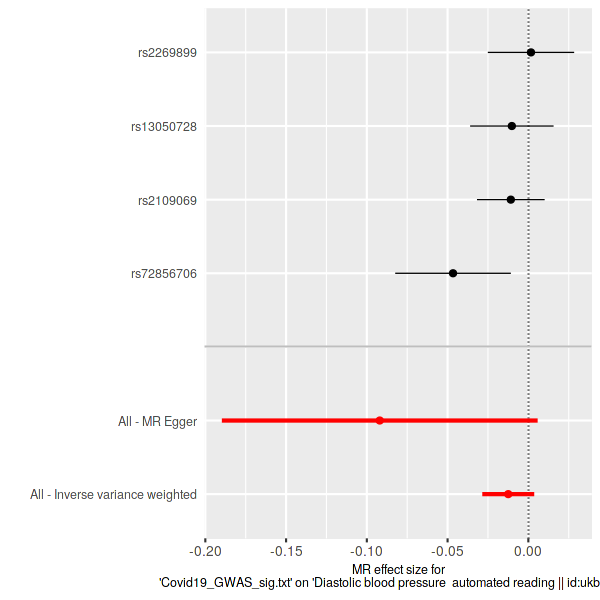


C)


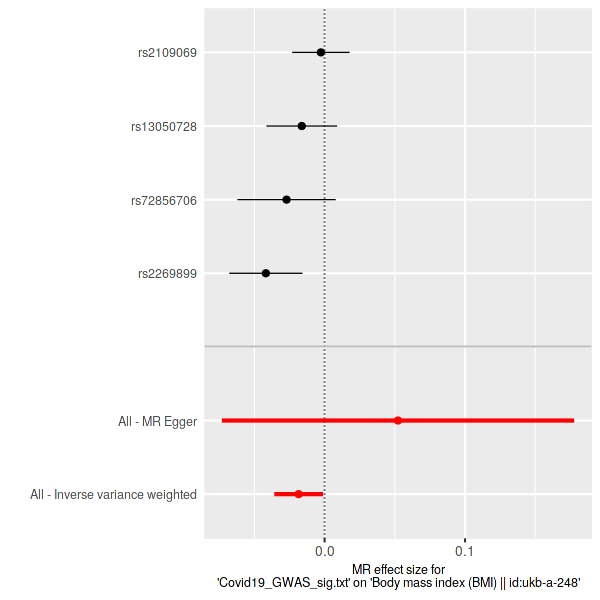


D)


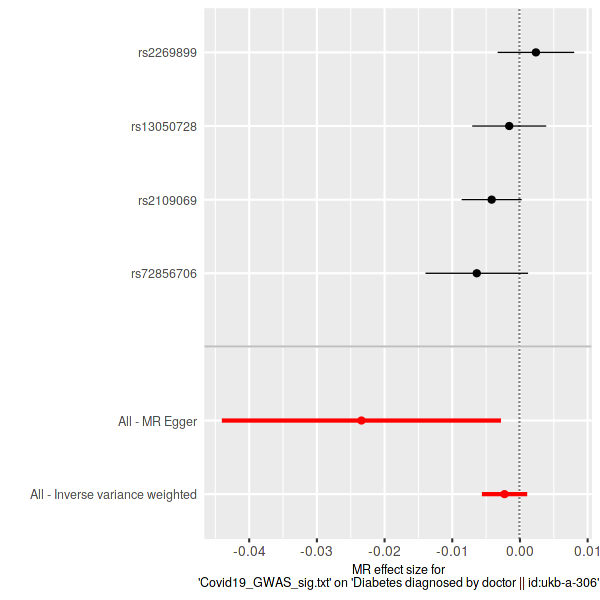


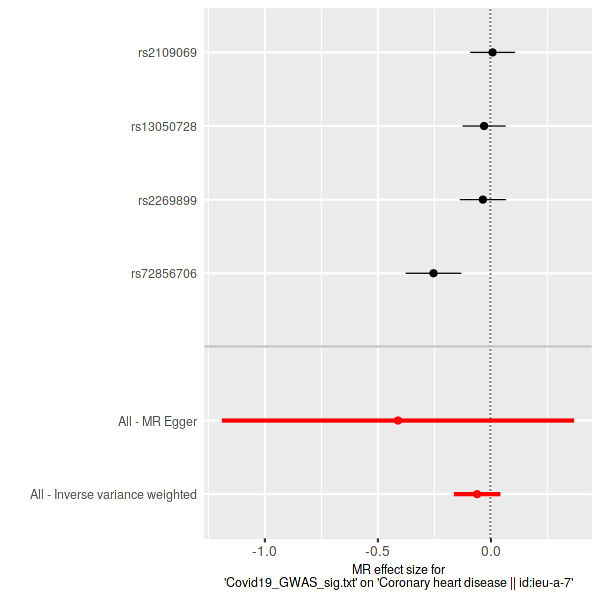
E)
